# Health Communication About Hospice Care in Chinese Media: Digital Topic Modeling Study

**DOI:** 10.2196/29375

**Published:** 2021-10-21

**Authors:** Qian Liu, Zequan Zheng, Jingsen Chen, Winghei Tsang, Shan Jin, Yimin Zhang, Babatunde Akinwunmi, Casper JP Zhang, Wai-kit Ming

**Affiliations:** 1 School of Journalism and Communication National Media Experimental Teaching Demonstration Center Jinan University Guangzhou China; 2 Department of Communication University of Albany State University of New York New York, NY United States; 3 Department of Public Health and Preventive Medicine School of Medicine Jinan University Guangzhou China; 4 School of Materials and Energy University of Electronic Science and Technology of China Chengdu China; 5 Maternal-Fetal Medicine Unit Department of Obstetrics and Gynecology Brigham and Women’s Hospital Boston, MA United States; 6 Center for Genomic Medicine, Massachusetts General Hospital Harvard Medical School Harvard University Boston, MA United States; 7 School of Public Health The University of Hong Kong Hong Kong China; 8 Department of Infectious Diseases and Public Health Jockey Club College of Veterinary Medicine and Life Sciences City University of Hong Kong Hong Kong China

**Keywords:** health communication, hospice care, mass media, China, topic modeling, communication, media, model, hospice, end-of-life, misconception, health information, news

## Abstract

**Background:**

Hospice care, a type of end-of-life care provided for dying patients and their families, has been rooted in China since the 1980s. It can improve receivers’ quality of life as well as ease their economic burden. The Chinese mass media have continued to actively dispel misconceptions surrounding hospice care and deliver the latest information to citizens.

**Objective:**

This study aims to retrieve and analyze news reports on hospice care in order to gain insight into whether any differences existed in heath information delivered over time and to evaluate the role of mass media in health communication in recent years.

**Methods:**

We searched the Huike (WiseSearch) news database for relevant news reports from Chinese mass media released between 2014 and 2019. We defined two time periods for this study: (1) January 1, 2014, to December 31, 2016, and (2) January 1, 2017, to December 31, 2019. The data cleaning process was completed using Python. We determined appropriate topic numbers for these two periods based on the coherence score and applied latent Dirichlet allocation topic modeling. Keywords for each topic and corresponding topics’ names were then generated. The topics were plotted into different circles, and their distances on the 2D plane was represented by multidimensional scaling.

**Results:**

After removing duplicated and irrelevant news articles, we obtained a total of 2227 articles. We chose 8 as the suitable topic number for both study periods and generated topic names and associated keywords. The top 3 most reported topics in the first period were *patient treatment*, *hospice care stories*, and *development of health care services and health insurance*, accounting for 18.68% (178/953), 16.58% (158/953), and 14.17% (135/953) of the collected reports, respectively. The top 3 most reported topics in the second period were *hospice care stories*, *patient treatment*, and *development of health care services*, accounting for 15.62% (199/953), 15.38% (15.38/953), and 14.27% (182/953), respectively.

**Conclusions:**

Topic modeling of news reports gives us a better understanding of the patterns of health communication about hospice care by mass media. Chinese mass media frequently reported on hospice care in April of every year on account of a traditional Chinese festival. Moreover, an increase in coverage was observed in the second period. The two periods shared 6 similar topics, of which *patient treatment outstrips hospice care stories* was the most reported topic in the second period, implying the humanistic spirit behind the reports. Based on the findings of this study, we suggest stakeholders cooperate with the mass media when planning to update policies.

## Introduction

Hospice care is the end-of-life care for patients with a terminal illness or critical condition and limited life expectancy, provided usually in their last 6 months of life or less. It also involves support and education about death provided for the patients’ families [1]. Hospice care aims to help these patients die peacefully, comfortably, and with dignity by controlling various types of pain and other symptoms [2]. In most situations, hospice care is offered at home, but it can also be offered in private or public health facilities, such as hospitals, specialized hospice facilities, or nursing homes. It is usually provided by a comprehensive unit consisting of nurses, social workers, home health aides, chaplains, volunteers, physicians, and hospice medical management or directors [3]. The first modern hospice was founded by Cicely Saunders, a British nurse, in 1967. Her experience of care for a dying refugee motivated her to build up a caring environment where patients could spend their last days [4]. In mainland China, the establishment of the first mainland China hospice hospital, Songtang Care Hospital, founded in 1987 in Beijing, marks the milestone of hospice care [5]. Since then, many policies have been established to promote hospice care in China. Several other hospice facilities were built, and various research studies on hospice care have also been conducted.

Hospice care is essential as it has been proven to improve the quality of life for patients [6]; ensure lower medical cost [7]; and possibly reduce the risk of death among surviving bereaved spouses, close relatives, or loved ones [8]. There is a huge demand for hospice care in China. Approximately 4.3 million new cancer cases and 2.9 million new cancer deaths were reported in 2018 in China [9]. These patients experience pain, anorexia, fatigue, myalgia, and labored breathing, among other issues [10]. Moreover, the number of patients with chronic diseases is also rising as the proportion of the elderly is increasing. According to the National Bureau of Statistics of China, it is estimated that over 250 million people are aged 60 years and above, accounting for 18.1% of the entire population [11]. Most patients also suffer from ongoing diseases and fear of death, and their families need psychological and spiritual encouragement. However, hospice care lacks adequate public awareness and social acceptance in China. A cross-sectional investigation conducted in two hospitals in Beijing among outpatients and family members revealed that less than 20% of them know or have even heard of hospice care [12]. Not just the patients and their families, but the medical staff alike, have little acquaintance with hospice care. Another cross-sectional study found that only about half of the health care providers know about hospice care, and they consider their overall knowledge of end-of-life care as inadequate [13]. In addition, the society—influenced by the traditional Chinese culture—prioritizes life-prolonging measures rather than improvement of life quality even in cases where the disease is impossible to cure, and death cannot be avoided [5]. Owing to this flawed notion, the family members refuse hospice care because they are afraid of being accused of not being a good son or daughter, as according to the Chinese culture and traditions, they are required to remain devoted to their parents until the end of their lives. Furthermore, many people misunderstand the system and believe that they will be abandoned if they received hospice care [5]. Therefore, the policymakers and health providers have an obligation to break the impediments to providing quality hospice care and further improve the public’s knowledge of hospice care.

The Chinese mass media channels have constantly published news reports related to hospice care, which aroused widespread discussion in China. These reports break the taboo of talking about death and dying, and actively engage in public advocacy and education on this topic. However, few studies have focused on the role that mass media plays in communicating the concept of hospice care. Therefore, in this study, we aim to collect the news reports pertaining to hospice care from major Chinese media portals and analyze them to determine the patterns of health communication through mass media. Multimodal data modeling can aggregate numerous information from different resources. To deal with multimodal data, topic modeling, a machine learning method that arranges unstructured data structurally in conformity with latent themes [14], was applied. By this means, we could investigate what health information about hospice care has been conveyed by the mass media to the public and whether it has changed over time.

## Methods

### Data Collection

“Hospice care” has different references in Chinese, including “gu xi hu li,” “an ning hu li,” “lin zhong guan huai,” and “an ning liao hu.” These terminologies are used interchangeably, and their corresponding Chinese characters are shown in Multimedia Appendix 1. We searched the Huike (WiseSearch) new database with these keywords. Founded in 1998, the Huike database is the leading expert Chinese media content database for integrating a massive number of authentic news reports from the Greater China region. It provides exclusive access to comprehensive Chinese news articles, with an average of over 96 million news items added daily from about 1600 print media sources and more than 50,000 internet media sources [15]. We restricted our collected sample to articles published by newspapers of mainland China. To identify whether any information changed over time, we collected Chinese news articles published in two different periods: (1) January 1, 2014, to December 31, 2016, and (2) January 1, 2017, to December 31, 2019. We specifically set 3-year periods because if we collect reports over long periods, latent Dirichlet allocation (LDA) may extract very broad topics, whereas we may not see the health information change if we check reports published in two adjacent short periods. We started our study in late 2020; therefore, we did not include the reports published in 2020 in our sample.

After collecting data, we used topic modeling to obtain useful information from the collected news reports. Topic modeling has been applied in various fields, such as text mining [16], psychology [17], and medicine [18], for data mining. In this study, we used LDA, one of the most well-known topic modeling forms to perform text analysis. LDA is a 3-level hierarchical Bayesian model for modeling text corpora. It assumes that documents, a sequence of random words, can be reflected as random mixtures over latent topics. Each of these topics is also represented by a probabilistic distribution over words [19]. By using Gibbs sampling, a method to estimate the marginal distributions of interested variables, the LDA model can determine the topics among the data pool [20].

### Processing

A total of 2227 articles were included, of which 953 articles were published between January 1, 2014, and December 31, 2016. The remaining 1274 reports were published between January 1, 2017, and December 31, 2019.

**Figure 1:**
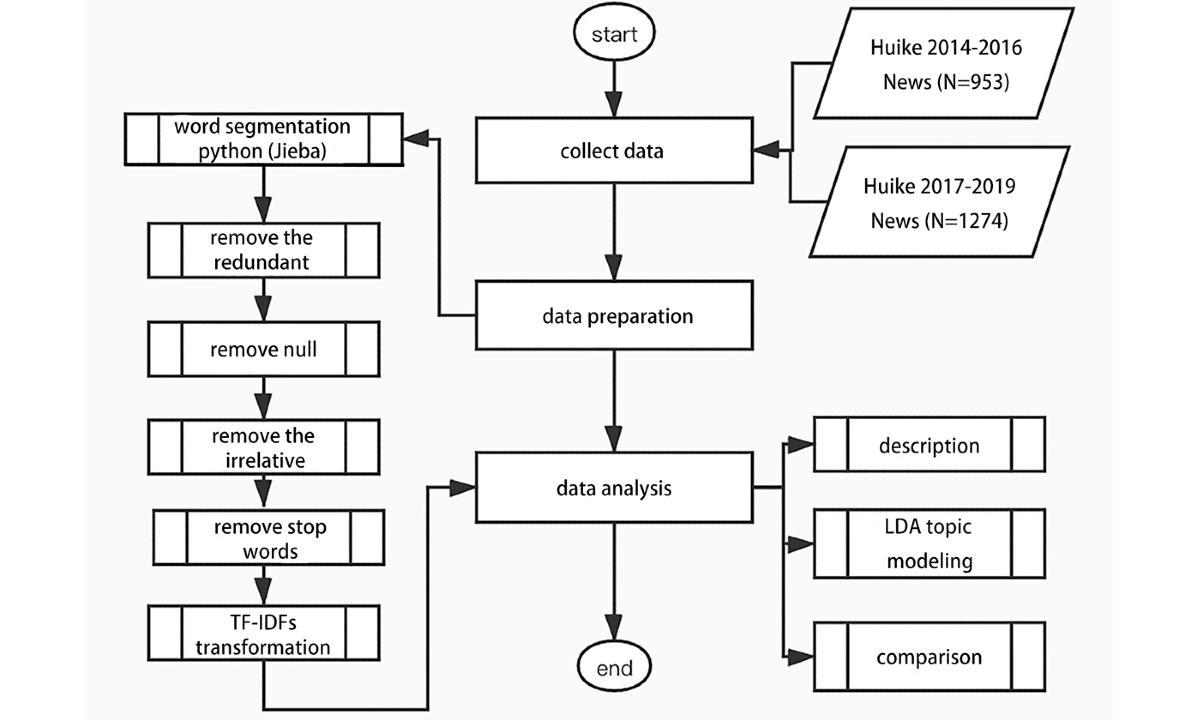
Data processing chart. LDA: latent Dirichlet allocation; TF-IDFs: term frequency-inverse document frequencies.

Data preparation was conducted before we applied the LDA. The process of data preparation is illustrated in Figure 1. Python 3.0 (Python Software Foundation) was used to perform data cleaning, and Python package Jieba was used to conduct word segmentation [21,22]. Redundant and null data were removed, and irrelative data were also excluded from our study. Next, common Chinese stop characters, such as “a,” “of,” and “ten” were removed (see Multimedia Appendix 1). A document-term matrix was established, and term frequency-inverse document frequency, a numerical statistic to show the significance of a word to an article in a corpus, was applied for data processing [23].

**Figure 2:**
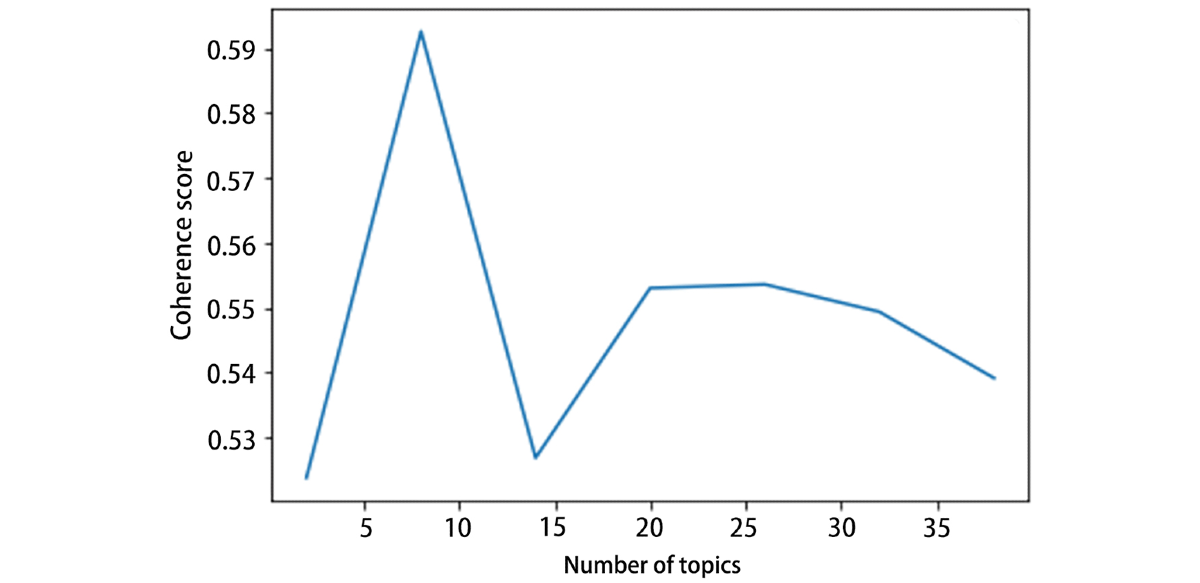
Coherence score for different topic numbers (2014-2016).

**Figure 3:**
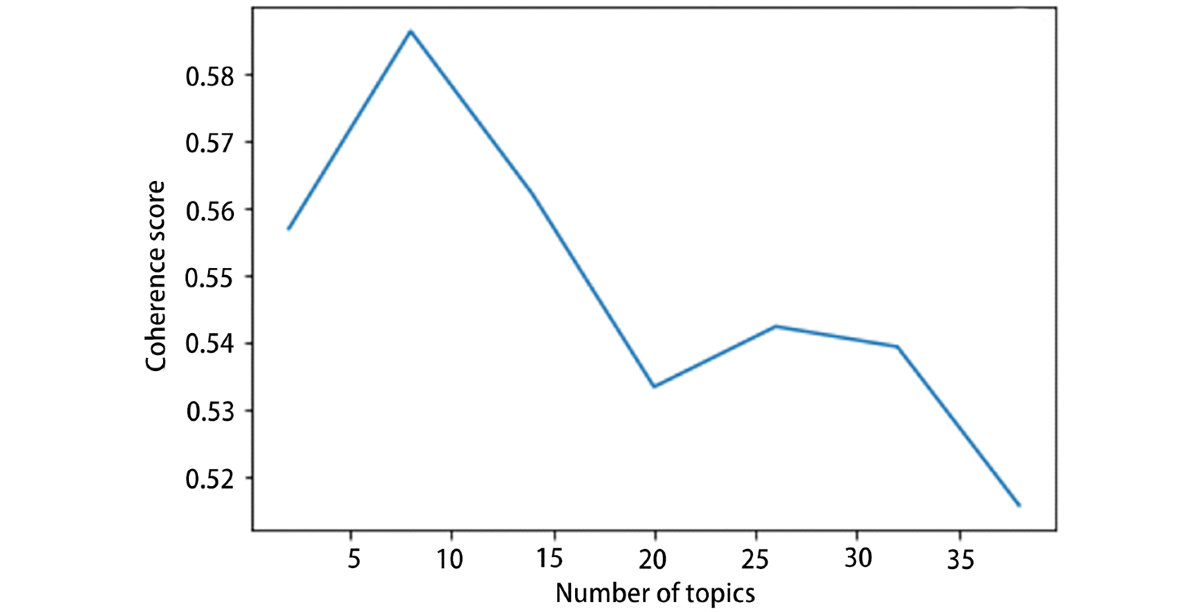
Coherence score for different topic numbers (2017-2019).

The selection of the LDA topic number is significant. Inclusion of too many topics will cause difficulty in interpretation and subjective validation, whereas too few topics can make the selected topics too broad [14]. To seek the optimum number of topics that LDA needs to extract from the collected news, several evaluation metrics were considered. Topic coherence is a qualitative method to score a topic’s coherence [24]. It measures the degree of semantic similarity between the top words associated with this topic. The generated topic is regarded as coherent if all or most of these keywords support each other, making it easier to interpret the outcomes. In the process of mathematical modeling, each top keyword of a single topic is converted into a context vector using word co-occurrence. The value of topic coherence is computed as the average of cosine values between every two context vectors [25]. In this study, the coherence model from Gensim (RARE Technologies Ltd)—the Python package for natural language processing—was used to calculate the coherence value [26]. Figures 2 and 3 show that the coherence value reached the highest score when the number of topics reached approximately 8. Thus, we chose 8 as our number of topics and set λ=1 to employ the LDAvis tool [19].

Each topic content was generated based on its associated set of keywords. However, no matter how advanced the statistical measures are, the output is not guaranteed to be interpretable because of the complexity of the language [27]. Therefore, we added manual interpretation to analyze the topics. Topics were also named according to the corresponding keywords to illustrate the topics. Tables 1 and 2 show the names of the topics and their keywords across the two periods evaluated.

**Figure 4:**
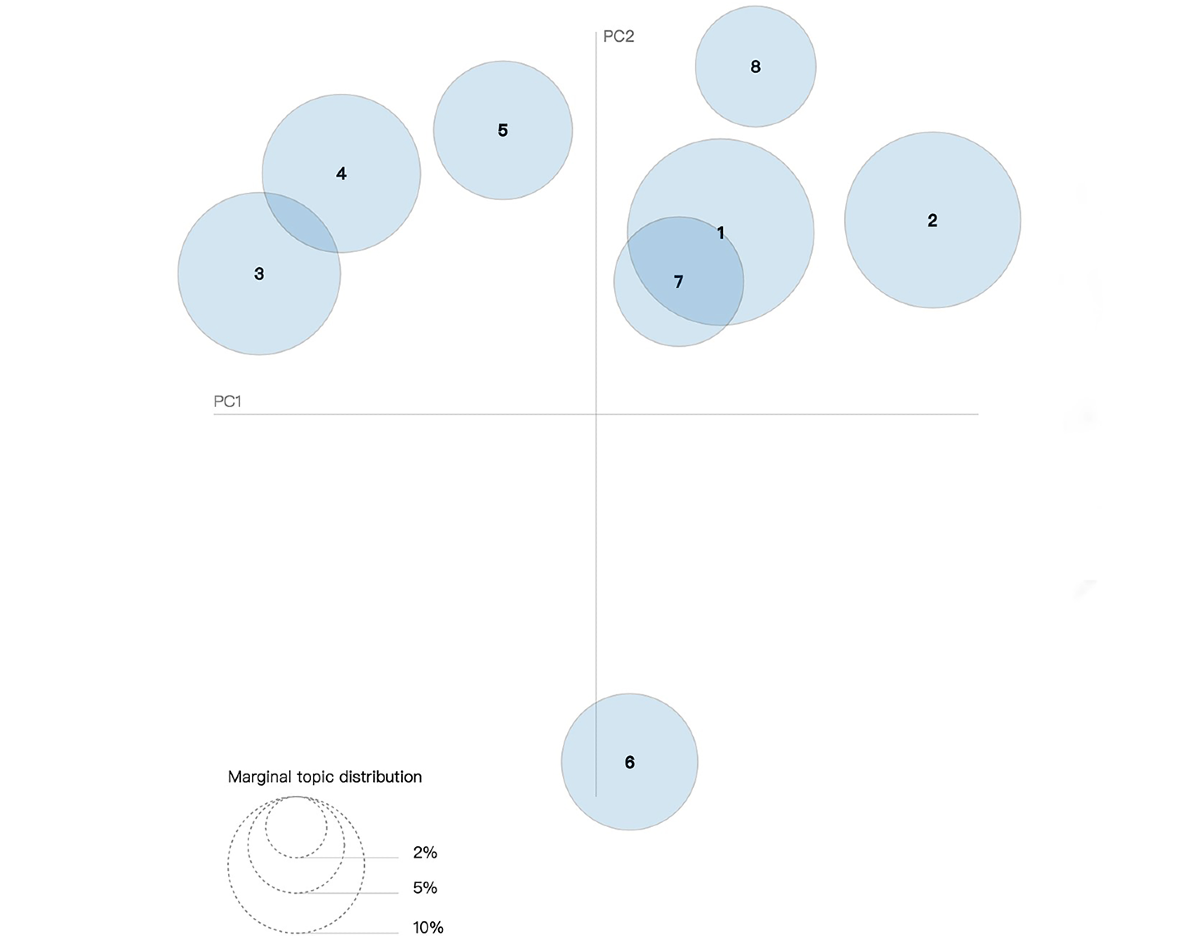
Intertopic distance map (via multidimensional scaling) for 2014-2016. PC: principal component.

**Figure 5:**
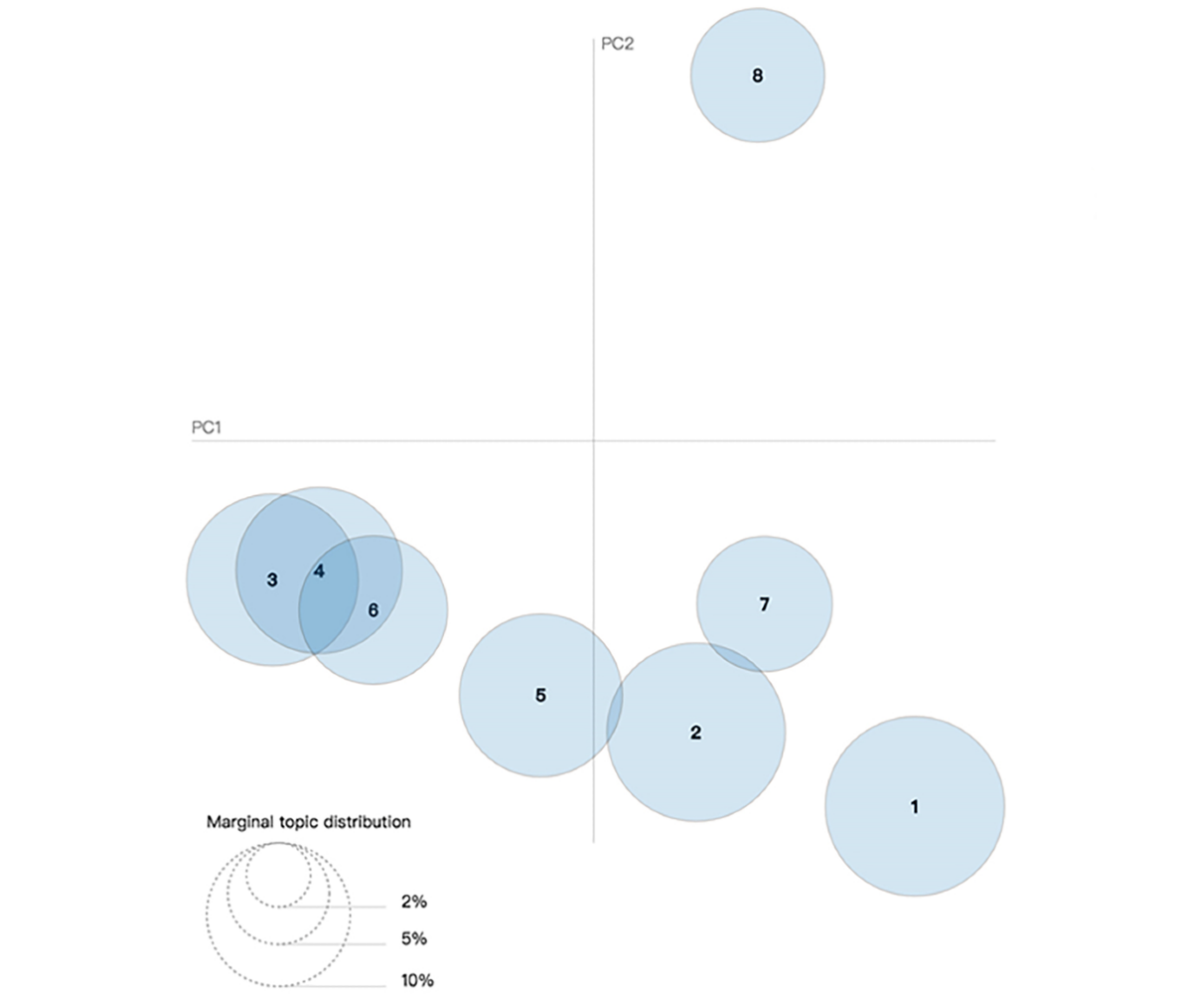
Intertopic distance map (via multidimensional scaling) for 2017-2019. PC: principal component.

We also plotted our topics as circles on a 2D plane (Figures 4 and 5) to determine their relationship. The centers of the circles were determined by the calculated distance between topics [19].

**Table 1:** Topic classification and keywords for 2014-2016.

Topic order	Topic name and keywords	News reports (N=953), n (%)^a^
Topic 1	Patient treatment Keywords: hospice care, patient, the sick, hospital, treatment, life, relative, end of life, death, care, inpatient ward, need, pain, medical treatment, mentality, finally, dignity, doctor, cancer, relieve, terminal stage, tumor, accept, nursing, proceed, provide, disease, quality, palliative, at present	178 (18.68)
Topic 2	Hospice care stories Keywords: the elderly, one, last, job, life, children, already, hope, mother, doctor, know, people, accompany, at present, think, nurse, everyday, father, always, leave, look after, see, once, time, pass away, inpatient ward, tell, son, face	158 (16.58)
Topic 3	Development of health care services and health insurance Keywords: service, development, society, medical care, peaceful, health, medical institution, institution, job, medical, establish, government, carry out, policy, construct, promote, health insurance, hygiene, community health, include, support, system, encourage, correlation, management, medical care and health, service center, improve, perfect, increase	135 (14.17)
Topic 4	Retirement and nursing home Keywords: pension, the elderly, service, nursing, institution, the aged, agedness, medical, center, medical treatment and aged care, rehabilitation, combination, hospital, community, provide, household, retirement home, mode, bed, life, journalist, look after, long-term, above, at present, disability, health, demand, profession, construction	129 (13.54)
Topic 5	Community services and social welfare activities Keywords: service, hospice care, volunteer, social worker, program, community, activity, carry out, care, social welfare, volunteerism, journalist, organization, profession, spirit, team, mentality, provide, social work, concern, job, family, participate, proceed, compassion, service center, establish, help, China	98 (10.28)
Topic 6	Huike platform statement Keywords: content, need, represent, integrity, author, website, in charge of, snapshot, check, only for, statement, connect, search, irrelevant, webpage, click, information, page, original text, instant, linkage, index, free of charge, Huike (WiseSearch), life, offspring, vacation for caring parents, China, society, culture	95 (9.97)
Topic 7	Hospice ward in the hospital Keywords: hospital, hospice care, death, journalist, citizen, express, one, enterprise, housing state, education, already, at present, enter, correlation, think, company, America, Hong Kong, around, family, proceed, objection, plan, construction, discover, this year, problem, brilliant, economy, consider	86 (9.02)
Topic 8	Voluntary service Keywords: service, volunteer, patient, cancer, terminal stage, hospice care, hospice, hospital, provide, establish, journalist, free of charge, poverty, family, president, life, the, visit, tailor, corpse, nationwide	74 (7.78)

^a^The total percentage is not 100% because of automatic rounding when exporting the results.

**Table 2:** Topic classification and keywords for 2017-2019.

Topic order	Topic name and keywords	News reports (N=953), n (%)^a^
Topic 9	Hospice care stories Keywords: the elderly, life, hospice care, one, death, final, volunteer, job, accompany, inpatient ward, living, hope, relatives, tell, see, leave, face, child, nurse, already, everyday, dying, journalist, livelihood, volunteer, always, know, time, family member	199 (15.62)
Topic 10	Patient treatment Keywords: patient, treatment, the sick, relative, cancer, inpatient ward, doctor, terminal stage, suffering, tumor, medical staff, mentality, life, final, nursing, pain, nurse, palliative, dignity, medical, disease, proceed, condition of disease, hospice, relieve, team, look after, provide, admission	196 (15.38)
Topic 11	Development of health care services Keywords: health, service, nursing, development, construction, medical, promote, nurse, job, management, establish, society, increase, primary, strengthen, medical institution, complete, implement, carry out, regime, family doctor, industry, encourage, hygiene, push forward, capacity, emphasis, increase, diagnosis and treatment, system	182 (14.27)
Topic 12	Retirement home Keywords: pension, service, institution, the elderly, nursing, combination of medical treatment and aged care, the aged, combination, medical, community, rehabilitation, provide, agedness, hospital, household, mode, center, family, disability, look after, bed, life, retirement home, management, above, carry out, journalist, construction, health, countryside	171 (13.42)
Topic 13	Lin zhong guan huai service (hospice care service in Chinese) Keywords: hospice care, service, hospital, society, demand, end of life, medical, problem, death, life, China, at present, development, profession, our country, education, suggestion, think, express, some, one, social worker, government, correlation, enterprise, care, need, important, provide, support	164 (12.95)
Topic 14	An ning liao hu service (hospice care service in Chinese) Keywords: peaceful, care, service, patient, center, pilot program, hospital, job, carry out, provide, country, nationwide, service center, look after, dignity, life, hygiene, terminal stage, care, community health, impatient ward, mentality, agedness, humanistic, medical institution, pilot work, institution, disease, journalist, establish	136 (10.68)
Topic 15	Social welfare activities Keywords: activity, society, volunteer, social welfare, family, compassion, job, community, program, volunteerism, culture, concern and love, China, life, learn, child, help, one, spirit, organization, become, student, charity, participate, Shanghai, team, people, entrepreneurship programs, join, world	113 (8.95)
Topic 16	Huike platform statement Keywords: content, represent, in charge of, integrity, author, website, snapshot, statement, check, connection, instant, only for, click, search, free of charge, irrelevant, original text, link, information, page, index, Huike (WiseSearch), webpage, need, profession, journalist, hospital, the newspaper, center, correspondent	111 (8.71)

^a^The total percentage is not 100% because of automatic rounding when exporting the results.

## Results

We categorized our collected sample of news articles into 8 topics for each period (ie, January 1, 2014, to December 31, 2016, and January 1, 2017, to December 31, 2019) by applying LDA topic modeling (Tables 1 and 2). These two periods share several similar topics, among which *patient treatment* and *hospice care stories* emerged as the two most popular topics. Topic 3 (*development of health care services and health insurance*, n=135) and topic 11 (ie, *development of health care services*, n=182), ranked as the third most popular topic, both accounting for approximately 14%. Topic 4 (*retirement and nursing home*, n=129) and topic 12 (*retirement home*, n=171) accounted for over 13%.

Figures 4 and 5 present the overall view of our topic model. Each figure shows 8 circles that represent various topics. We can calculate the overall prevalence by computing areas of all circles. Intertopic distances are represented by multidimensional scaling on a 2D plane [28]. The principal components PC1 and PC2 represent the transverse axis and longitudinal axis, respectively.

**Figure 6:**
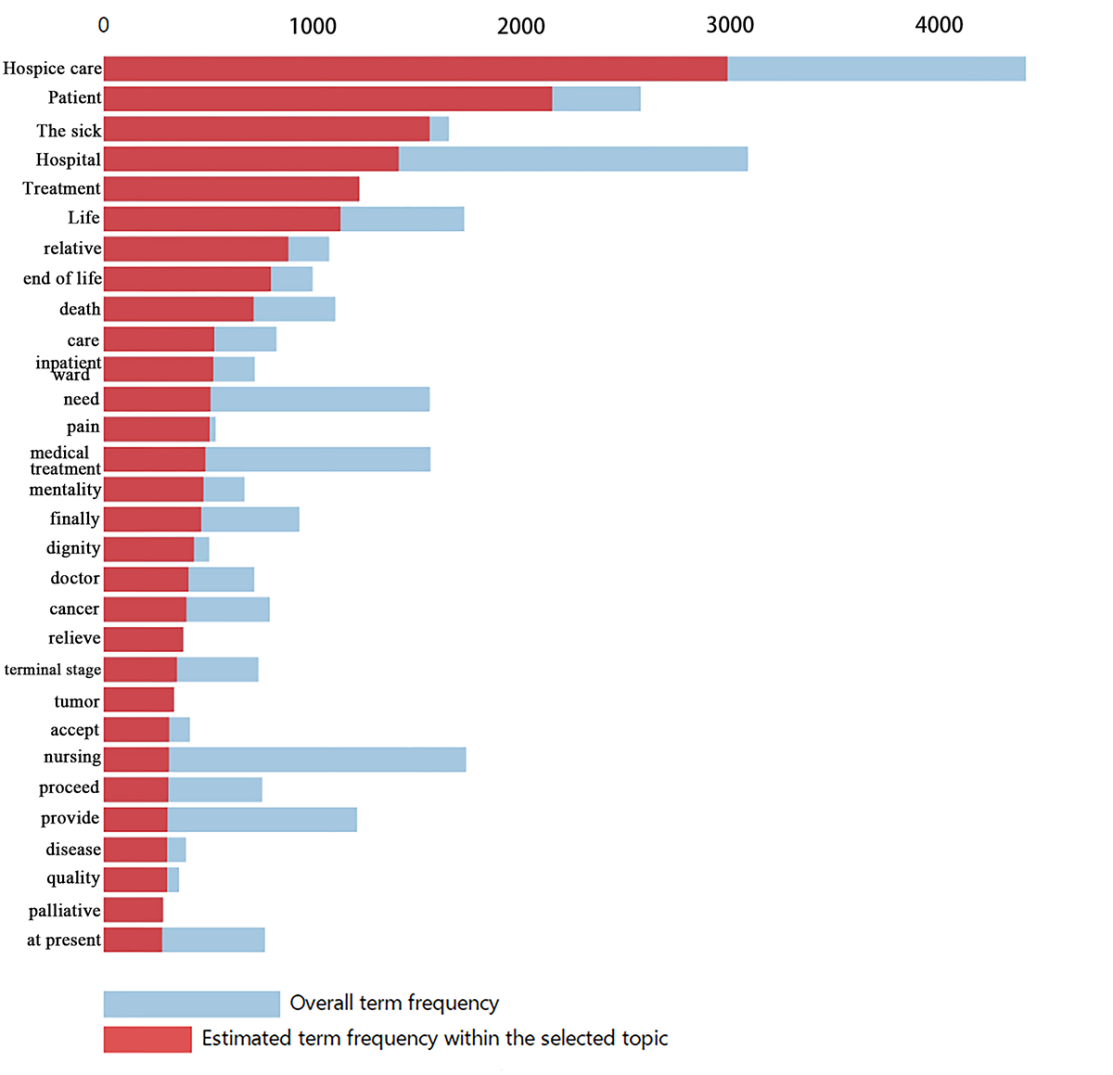
Top 30 most relevant terms for topic 1 (18.68% of all news reports). Saliency (term w) = frequency (w) * [sum_t p(t | w)/p(t)] for topics t; see [29]. Relevance (term w | topic t) = λ * p(w | t)/p(w); see [30].

**Figure 7:**
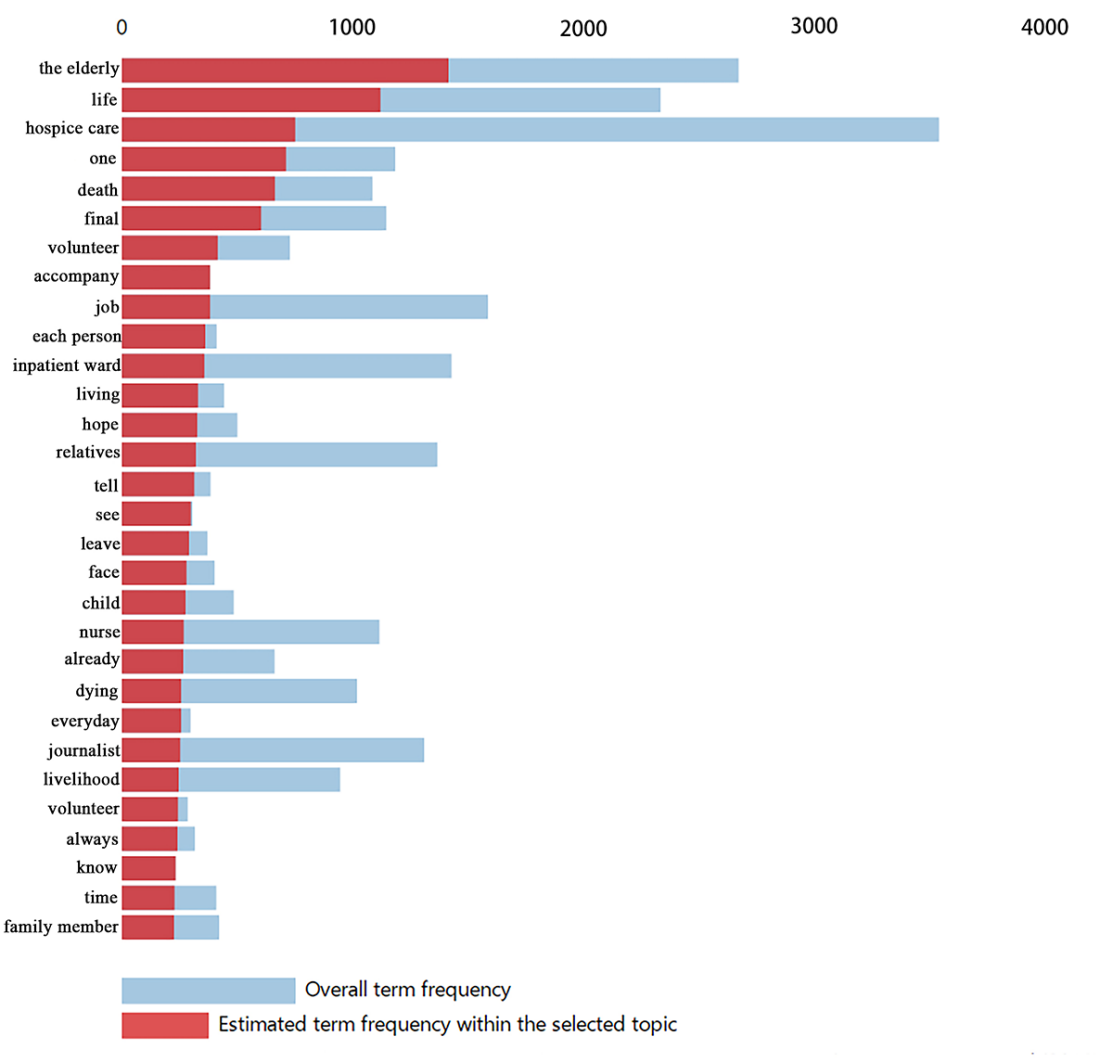
Top 30 most relevant terms for topic 9 (15.62% of all news reports). Saliency (term w) = frequency (w) * [sum_t p(t | w)/p(t)] for topics t; see [29]. Relevance (term w | topic t) = λ * p(w | t)/p(w); see [30].

Figures 6 and 7 present the top 30 relevant terms for topics 1 and 9, respectively. These two topics had the highest proportion of news articles in their respective time periods; therefore, we present them as an example for demonstration. The word frequency distribution is relative to the full corpus by the system. The blue bar presents the overall term frequency, and the red bar presents the estimated frequency of a specific topic. With regard to topic 1, Chinese mass media preferred to talk about what treatment the patients can receive at the end of life. Using this approach, as illustrated in the literature, we could interpret the content of a topic [29,30].

**Figure 8:**
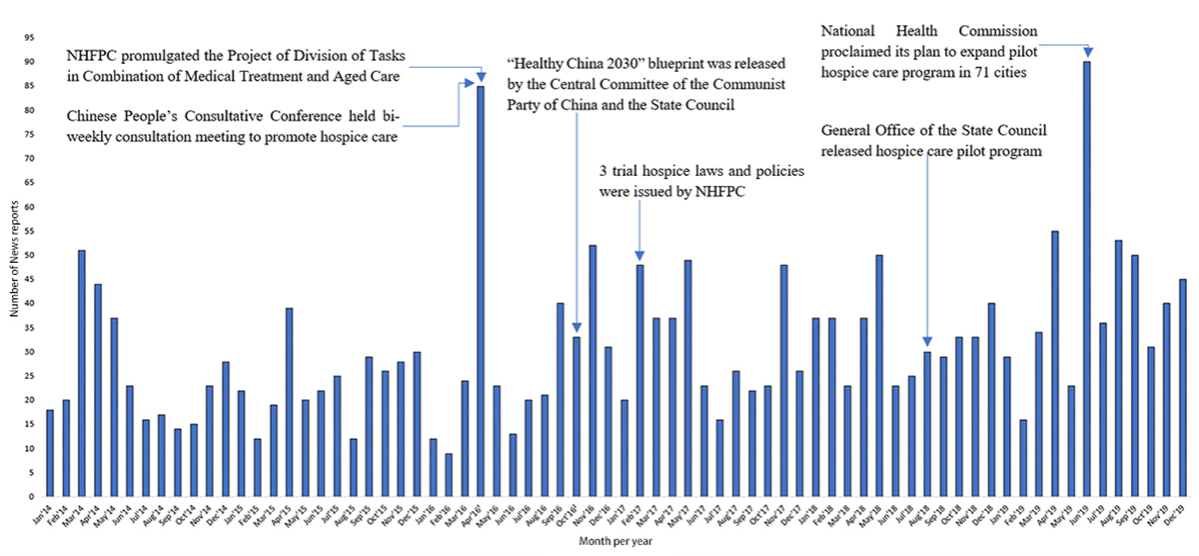
Time series of news streams with corresponding laws and polices during 2014-2019. NHFPC: National Health and Family Planning Commission of the People’s Republic of China.

Figure 8 shows the number of hospice care news reports published over time. The number of news articles peaks and wanes across different months. In the first period, the total coverage reached 953, and the highest monthly coverage was 85 news reports. In the second period, the number of total relevant news reports was 1274, and the monthly coverage peaked in June 2019. Table 3 shows Chinese laws and policies about hospice care and their release dates.

**Table 3:** Chinese laws and policies about hospice care.

Date	Laws and policies
April 7, 2016	The National Health and Family Planning Commission of the People’s Republic of China (NHFPC, the predecessor of National Health Commission) promulgated the Project of Division of Tasks in Combination of Medical Treatment and Aged Care to integrate hospice care into the elderly care system [31].
April 21, 2016	Chinese People’s Consultative Conference (a political advisory body of the People’s Republic of China and a central part of the Chinese Communist Party’s United Front system) held biweekly consultation meeting to promote hospice care [32].
October 25, 2016	“Healthy China 2030” blueprint was released by the Central Committee of the Communist Party of China (a political body that comprises the top leaders of the Chinese Communist Party) and the State Council to strengthen the construction of hospice care institutions [33].
February 9, 2017	Hospice Care Center Basic Standards (trial), Hospice Care Center Management Standardization (trial), and Hospice Care Practice Guideline (trial) were issued by the NHFPC [34,35].
August 28, 2018	General Office of the State Council released Major Projects for Deepening Medical and Health System Reform in the Second Half of 2018, including the hospice care pilot program [36].
June 10, 2019	The National Health Commission proclaimed its plan to expand pilot hospice care program in 71 cities [37].

Tables 4 and 5 show the top 10 productive media sources with regard to publication of news reports on hospice care. Local and national newspapers were all engaged. Table 4 shows that Xin’an Evening News (Digital News) had the highest coverage during 2014-2016, with 21 (11.67%) news reports, followed by Xinmin Evening News and China News service (11/953, 11.11%). As shown in Table 5, the People’s Political Consultative Daily (Digital News) was the most active mass media in the second study period, with 39 (23.07%) relevant articles published. It was during the press window of policies and viewpoints of the Chinese People’s Political Consultative Conference wherein there was considerable reporting about the biweekly consultation meetings held by the Chinese People’s Political Consultative Conference to promote hospice care [32]. In addition, 18 (10.65%) news reports were published in the Middle-Aged Times.

**Table 4:** The most represented media sources for news reports collected during 2014-2016 (N=953).

Media sources	News reports, n (%)
Xin’an Evening News (Digital News)	21 (11.67)
Xinmin Evening News	20 (11.11)
China News Service	20 (11.11)
Qilu Evening News (Digital News)	19 (10.56)
Shanxi Evening News	18 (10.00)
Sanjin Metropolis Daily	18 (10.00)
Youth Daily	17 (9.44)
Shaanxi Daily	16 (8.89)
Workers' daily	16 (8.89)
Shanxi Daily (Digital News)	15 (8.33)

**Table 5:** The most represented media sources for news reports collected during 2017-2019 (N=1274).

Media sources	News reports, n (%)
People’s Political Consultative Daily (Digital News)	39 (23.07)
Middle-aged Times	18 (10.65)
Yantai Daily	17 (10.06)
Qilu Evening News (Digital News)	17 (10.06)
Shanxi Daily (Digital News)	17 (10.06)
Wuhan Evening News	16 (9.47)
China News Service	12 (7.10)
Xinmin Evening News	12 (7.10)
Zhongshan Commercial Daily	11 (6.51)
Workers' daily	10 (5.92)

## Discussion

### Principal Findings

Hospice care has received much attention in China in the context of aging of the Chinese population. Topic modeling is a new method that helps to shed light on what health information has been delivered by the mass media. In this study, we observed that the number of reports about hospice care in the second study period slightly increased compared to the first period, indicating Chinese mass media had paid more attention to this topic. We also found that mass media tended to focus on hospice care in April of every year (Figure 8). Of all the monthly news about hospice care published between January 1, 2014, and December 31, 2016, coverage peaked in April (168 reports published). Although the proportion of news reports in April decreased in the second study period, it still accounted for 10.11% of the collected reports, ranking as the second month with the highest number of reports. This is likely because the Tomb-Sweeping Day, a traditional Chinese festival, falls on April 4 to 6 every year. On this day, people in China usually clean up the graves of their ancestors and deceased relatives to show their grief. As a result, mass media channels strategically chose this timeline to focus on hospice care and discuss the contemporary view of life and death, potentially making this concept more acceptable for the general public.

Supportive laws and policies are indispensable for the optimal development of hospice care services [38], and the promulgation of laws and policies often contribute to an increase of coverage. For example, relevant laws and policies were released in February 2017 [34,35] and June 2019 [37], and a considerable increase in articles about hospice care was witnessed in these 2 months (Figure 8). In some cases, however, mass media did not leverage the opportunity to broadcast new policies. For instance, in October 2016, the “Healthy China 2030” blueprint was issued [33], while there were few news reports about hospice care in October. Therefore, if the government wants to ensure the citizens are aware of the latest policies about hospice care, the mass media should be utilized to disseminate these policies.

In the second period, topic 9 (*hospice care stories*) overtook topic 10 (*patient treatment*) as the top news topic. Although both news topics focused on what happened after hospice care was provided to patients, reports pertaining to the topic *patient treatment* tended to follow a well-established pattern of reporting on the care patients received. Mass media channels conveyed health information by elucidating the role of the hospital, hospice, doctor, and family members in the process of treatment. The news articles addressing hospice care tended toward personal experience. These stories were narrated in a kind and vivid tone, which reflected the humanistic caring spirit in the reports. Mass media should continue to report hospice care with a humanistic caring spirit to make this topic more acceptable and promote health education well.

The development of health care services is also a major concern of the media. In the first period, the mass media linked the development of health care services with the development of health insurance, proclaimed the benefits to patients and medical institutions of the government’s promotion, and related system development, which stressed the importance of health insurance. However, most of the hospice care services are still not included in the national health insurance [39]. The high cost of hospice care is a barrier for most patients. In addition to describing the development of health care services, many articles related to topic 11 also introduced the concept of the family doctor, which was a new highlight of the development.

Retirement homes and nursing homes are important places to facilitate hospice care [40]. Coverage of topic 12 focused on the significance of these institutions. Furthermore, topic 4 covered multi-modeled cooperation between hospitals and retirement homes. In April 2016, the NHFPC enacted the Project of Division of Tasks in Combination of Medical Treatment and Aged Care to encourage this multi-modeled cooperation [31]. In the process of cooperation, an integral elderly care system consisting of daily life care, medical treatment, rehabilitation nursing, and hospice care, is expected to be built. It can address the imbalance between the huge need for hospice care and the lack of hospice institutions, as well as maximize the benefits of hospice care in the elderly care system.

In these two defined periods, social welfare activities also received wide coverage in the Chinese mass media. Topic 5 (*community services and social welfare activities*) emphasized that volunteers working in hospice care were significant to society, whereas topic 15 (*social welfare activities*) in the second period also referred to the participation of other social forces, such as student teams and entrepreneurship programs. Since the initial stage, the modern hospice care movement has received sustained support from volunteers who were devoted to eliminating the stigma of hospice care, raising funds, attracting seldom-heard communities, and caring for the patients and their families directly [41]. In China, there are plenty of nonprofit organizations engaged in hospice care promotion, including the Hospice Palliative Care Alliance of China Foundation [42] and the Chinese Association for Life Care [43]. The coverage of welfare activities can help readers get acquainted with the work of volunteers in hospice care and appeal to the public to take part in it, which contributes to the social moral. It is worth mentioning that topic 8 (*voluntary service*) also reported the involvement of volunteers in hospice care.

Two topics from the second period vary from the first period. These are topic 13 (*lin zhong guan huai service*) and topic 14 (*an ning liao hu service*). Although both “lin Zhong guan huai” and “an ning liao hu” both mean hospice care in Chinese, news articles on these two topics attached significance to different contexts. For example, the news reports on topic 13 underlined the role of the hospital and community. The effect of community engagement activities on hospice care development is recognized, and these activities were a priority for most hospices in the United Kingdom [44]. The Chinese government also called on the community to shoulder the responsibility for caring the elderly in its 5-year plan (2016-2020) on care for older adults [45]. Reporting community significance on hospice care can appeal to community members to join in these activities. Topic 14, however, raised the necessity to pay more attention to the psychological problems of patients.

### Limitations

Our study is one of the first to reveal the role of Chinese mass media in communicating health information about hospice care. Our study has some limitations. We searched the Huike (WiseSearch) news database for data collection. This database only includes text news articles. Therefore, we might have missed news content in the form of images and short videos through some new media platforms such as WeChat (the most popular instant messaging app in China) and TikTok (a popular video social media platform among the young generation). Moreover, the inherent limitations of LDA restricted some part of our study, as LDA is limited to the analysis of few articles or overly short articles [46]. Sentiment analysis is a helpful method to evaluate positive or negative attitudes in the text [47]; it would be favorable if we could adopt it to help us obtain more information about the Chinese mass media’s attitude toward hospice care.

### Conclusions

We investigated the information about hospice care disseminated through Chinese mass media during two specific periods by analyzing collected news reports using topic modeling. We conclude that the coverage of hospice care generally increases in April of every year, which is related to a special traditional Chinese festival. We propose that authorities and policymakers in China should cooperate with the mass media to propagate the latest hospice care policies. Two of the most popular topics surrounding hospice care are *patient treatment* and *hospice care stories*, of which hospice care stories have accounted for a greater proportion of reports in recent years, demonstrating the humanistic caring spirit adopted by the mass media while reporting on this public health issue. Development of health care services and retirement homes are also reported, while the emphasis of each topic changes with time.

## Appendix

Multimedia Appendix 1 Various references to "hospice care" in the Chinese language and their corresponding Chinese characters.

## Abbreviations

LDA: latent Dirichlet allocation
NHFPC: National Health and Family Planning Commission of the People’s Republic of China
PC: principal component
