# Supplementary material for: Health Communication About Hospice Care in Chinese Media: Digital Topic Modeling Study
Source: JMIR Public Health Surveill. 2021 Oct 21;7(10):e29375. doi: 10.2196/29375 (PMC8569548; doi:10.2196/29375)
Supplement: Multimedia Appendix 1 [file publichealth_v7i10e29375_app1.docx]

Hospice care in Chinese phonetic alphabets and their corresponding Chinese characters

| Chinese phonetic alphabets | Chinese characters |
| --- | --- |
| gu xi hu li | 姑息护理 |
| an ning hu li | 安宁护理 |
| lin zhong guan huai | 临终关怀 |
| an ning liao hu | 安宁疗护 |

These Chinese words are used interchangeably, with the same meaning as “hospice care”.

Stop words and their corresponding Chinese characters

| A | 一 |
| --- | --- |
| Of | 的 |
| Ten | 十 |
